# A Doubly Fmoc-Protected Aspartic Acid Self-Assembles into Hydrogels Suitable for Bone Tissue Engineering

**DOI:** 10.3390/ma15248928

**Published:** 2022-12-14

**Authors:** Katerina Petropoulou, Varvara Platania, Maria Chatzinikolaidou, Anna Mitraki

**Affiliations:** 1 Department of Biology, University of Crete, 70013 Heraklion, Greece; 2 Department of Materials Science and Technology, University of Crete, 70013 Heraklion, Greece; 3 Institute of Electronic Structure and Laser (IESL), Foundation for Research and Technology Hellas (FO.R.T.H), 70013 Heraklion, Greece

**Keywords:** fluorenyl methoxycarbonyl (Fmoc), self-assembly, amyloid fibrils, injectable hydrogel, composite, calcium ion binding, building block, single amino acid, osteogenesis, biomineralization

## Abstract

Hydrogels have been used as scaffolds for biomineralization in tissue engineering and regenerative medicine for the repair and treatment of many tissue types. In the present work, we studied an amino acid-based material that is attached to protecting groups and self-assembles into biocompatible and stable nanostructures that are suitable for tissue engineering applications. Specifically, the doubly protected aspartic residue (Asp) with fluorenyl methoxycarbonyl (Fmoc) protecting groups have been shown to lead to the formation of well-ordered fibrous structures. Many amino acids and small peptides which are modified with protecting groups display relatively fast self-assembly and exhibit remarkable physicochemical properties leading to three-dimensional (3D) networks, the trapping of solvent molecules, and forming hydrogels. In this study, the self-assembling fibrous structures are targeted toward calcium binding and act as nucleation points for the binding of the available phosphate groups. The cell viability, proliferation, and osteogenic differentiation of pre-osteoblastic cells cultured on the formed hydrogel under various conditions demonstrate that hydrogel formation in CaCl_2_ and CaCl_2_-Na_2_HPO_4_ solutions lead to calcium ion binding onto the hydrogels and enrichment with phosphate groups, respectively, rendering these mechanically stable hydrogels osteoinductive scaffolds for bone tissue engineering.

## 1. Introduction

The self-assembly of peptides allows new materials to be obtained through a bottom-up methodology [1], leading to the development of peptide-based building blocks (i.e., from dipeptides to amphiphilic block copolymers) [2]. These systems are favorable in biomedical applications, including bone and cartilage regeneration [3,4], being inspired by nature [5]. Peptide self-assembly is very often associated with the formation of amyloid fibrils, which have been found to be useful as smart materials for developing scaffolds for tissue engineering [5,6,7,8]. Amyloid fibrils are believed to behave as significant gelators for producing supramolecular hydrogels [9]. Hydrogels are networks with a high capacity to retain water, similar to biological tissues.

Hydrogels, as a non-invasive material form in biomedicine, attract increasing attention due to their tunable physicochemical properties and ECM-mimicking characteristics [10,11]. Amongst them, supramolecular injectable hydrogels are investigated in drug delivery, tissue regeneration, and 3D cell culture [12]. Hydrogels can be classified considering the type of cross-links (i.e., physical or chemical) and their ability to incorporate chemical agents and cells. Furthermore, these systems can be designed with endowed shape memory and stimuli-responsive properties and in situ gelling materials that are ideal for tissue regeneration as they can be injected and adapted to the form of tissue defects.

The bioactivity of specific bone substitutes and their potential to stimulate the natural healing process in a defect site can be increased by the creation of hydroxyapatite deposition on the scaffold [13,14]. This is due to the attachment of osteoprogenitor cells and osteoblasts to a mineralized matrix that allows osteoblasts to deposit the extracellular collagen that will form the collagen matrix. Calcium and phosphate ions are produced by the osteoblasts and are secreted in the environment. Thus, new mineralization can be achieved when the hydroxyapatite crystals are formed onto the polymer matrix [15], which significantly improves the cell activity and the reparative phase towards bone regeneration.

Previous studies with amyloid peptide-based hydrogels have shown their applicability in developing scaffolds that support cell attachment and spreading [6,8,16,17]. However, amyloid-based hydrogels have not been widely explored yet as scaffolds for bone tissue engineering applications [18,19,20,21]. The protecting N-terminal group fluorenyl methoxycabonyl (Fmoc) is a potent gelator of self-assembling peptides, leading to the formation of hydrogels that can support cell attachment and proliferation and, therefore, can be used as scaffolds for tissue engineering [22,23,24]. Even single amino acids, when attached to protecting groups, can form hydrogels that are suitable for applications such as drug encapsulation and delivery, the encapsulation of metal nanoparticles for antibacterial materials, etc. [25]. Taking into consideration the advantages of simple Fmoc-protected amino acids and peptides for hydrogel formation and applications [26], we opted to study the self-organization of the minimal, doubly protected aspartic acid Fmoc-Asp-OFm. To our knowledge, either oligopeptide [19,27,28,29] or mixtures of Fmoc-protected dipeptides with protected amino acids were used so far as self-assembling scaffolds for bio-mineralization [20]. In this work, we opted to study a monomolecular building block that combines both the potent hydrogelation propensities of two Fmoc moieties and the calcium-nucleating properties of a single acidic amino acid. Acidic amino acids (either aspartate or glutamate) containing self-assembling peptides are crucial for hard tissue engineering, as they create a biomimetic fibrous network that can nucleate calcium ions [28,29,30]. We aimed to create a biomimetic hydrogel matrix with calcium ion binding sites that are expected to act as nucleation points for phosphate anions. First, we used the Fmoc-Asp-OFm building block to create hydrogels that were formed under various conditions, including calcium chloride (CaCl_2_) and calcium chloride, with dibasic sodium phosphate (CaCl_2_-Na_2_HPO_4_) solutions. Moreover, we optimized the formation conditions to achieve stable hydrogels with favorable mechanical properties and characterized them physiochemically. Furthermore, we assessed the adhesion, proliferation, and osteogenic differentiation potential of pre-osteoblastic cells cultured on Fmoc-Asp-OFm-based hydrogels. Our results demonstrate that hydrogel formation in the CaCl_2_ and CaCl_2_-Na_2_PO_4_ solution leads to calcium ion binding on the hydrogels and enrichment with phosphate groups, respectively, rendering them mechanically stable osteoinductive scaffolds for bone tissue engineering.

## 2. Materials and Methods

### 2.1. Preparation of Fmoc-Asp-OFm Hydrogels

**Scheme 1:**
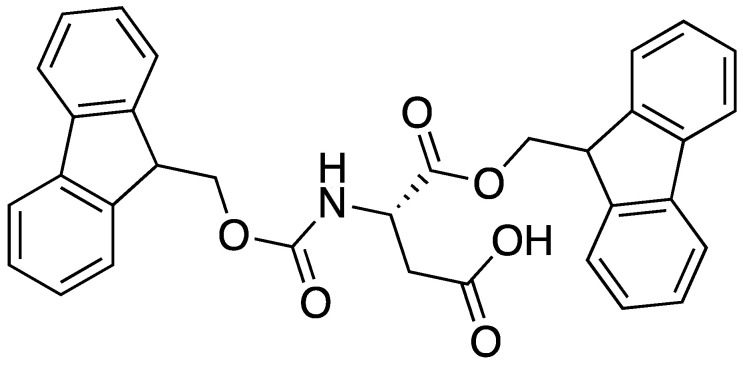
Chemical formula of the doubly protected aspartic residue (Asp) with fluorenyl methoxycarbonyl (Fmoc) protecting groups, Fmoc-Asp-OFm.

The doubly protected aspartic residue (Asp) with fluorenyl methoxycarbonyl (Fmoc) protecting groups (Fmoc-Asp-OFm) was purchased from Bachem (Bubendorf, Switzerland) in the form of lyophilized powder and had a degree of purity higher than 99%. The powder was dissolved in dimethyl sulfoxide (DMSO) at concentrations of 3 mg/mL which were followed by dilution into double-distilled water for the induction of self-assembly (control hydrogels). The chemical formula of Fmoc-Asp-OFm is presented in Scheme 1.

### 2.2. Ca^2+^ Mineralization of Hydrogels with Dibasic Calcium Phosphate and Binding of Phosphate

The powder was first dissolved in DMSO, and then a 7 mM CaCl_2_ solution was added and mixed with vortexing at a volume ratio of 2:8 DMSO/CaCl_2_. The solution was allowed to self-assemble for 24 h prior to its use in experiments. The hydrogel was cast in a cut syringe part, which was subsequently demolded in cell culture wells and thoroughly washed with water for 3 days, with changes every day until no calcium could be detected in the solution (Supporting Figure S1). This procedure ensured that only calcium ions attached by templating via electrostatic interactions on the negatively charged fibrillar network remained in the hydrogel. For the phosphate salt addition, a few drops of a 0.6 M solution of Na_2_HPO_4_ (pH 9) were subsequently poured over the hydrogel matrix. The composition and formation conditions of the hydrogels are presented in Table 1.

### 2.3. Field Emission Scanning Electron Microscopy (FESEM)

The surface morphology of the hydrogels was observed using a JEOL JSM 7000F SEM microscope operating at 15 kV. Before the observation, 10 μL of every sample solution was deposited on a circular glass and left to dry at room temperature. The samples were sputtered with a 10 nm thick layer of Au and were observed directly. The diameter of the mineralized nanoparticles was measured from FESEM images using the ImageJ software (National Institutes of Health, Bethesda, MD, USA). To determine the frequency distribution, a Gaussian (normal distribution) curve was plotted [31] by means of the software GraphPad Prism (GraphPad Software, San Diego, CA, USA) version 8. All samples were analyzed in triplicates.

### 2.4. Transmission Electron Microscopy (TEM)

TEM analysis was performed for the visualization of Fmoc-Asp-OFm self-assembly into nanofibers. The samples were prepared by depositing 8 μL of the sample on a carbon-coated grid with a diameter of 3 nm. After 2 min, the excess fluid was removed, and the same process was repeated with the negative stain. The samples were negatively stained with 8 μL 1% uranyl acetate. The samples were observed using a JEOL JEM-2100 (JEOL, Peabody, MA, USA) high-resolution transmission electron microscope with an accelerating voltage of 200 kV.

### 2.5. Congo Red Staining (CRS)

Each sample was mixed with a Congo Red assay solution (10 mM Congo Red, 2.5 mM NaOH in 50% ethanol) in a 6:1 ratio. A total of 10 μL of the sample was deposited on a glass coverslip and was allowed to dry. Then, it was examined with a Zeiss Stemi 2000-C stereoscope equipped with crossed polarizers.

### 2.6. Uniaxial Compression Testing

The mechanical properties of the Fmoc-Asp-OFm hydrogels were evaluated by uniaxial compression tests. Hydrogel samples with a diameter of 1.2 cm and a height of 2 cm were employed. The compressive stress–strain tests were performed by using an UniVert mechanical test system equipped with a 50 N sensor in the air at room temperature. The Fmoc-Asp-OFm hydrogels were prepared into cylindrical molds, left to self-assemble overnight, and tested after approximately 24 h. The measurements were performed in five replicates (n = 5). Compression loading was performed at a 5 mm/min deformation speed of up to 60% strain. The Young’s modulus of the samples was determined as the slope in the linear elastic deformation region of the stress–strain diagrams.

### 2.7. Alizarin Red Staining (ARS)

After the formation of the hydrogel with calcium, 1 mL of freshly prepared Alizarin Red S was added to the solution and incubated at room temperature for 30 min (40 mM ARS pH: 4.1–4.5). The dye was subsequently removed, and the hydrogels were washed several times with ddH_2_O. The hydrogels were observed microscopically for an orange-red color.

### 2.8. X-ray Diffraction Analysis (XRD)

XRD was used to evaluate the hydroxyapatite composition in the hydrogels. To perform this analysis, the hydrogels were first freeze-dried for 36 h for water removal and then milled to obtain a fine powder. Structural analysis of the InN films was performed by the triple axis in a high-resolution X-ray diffractometer (HR-XRD) BEDE D1 with Cu Kα radiation (λ = 1.5460 Å), operating at 45 kV and 40 mA, for an angle step of 0.0241° and 2θ range from 5 to 60°.

### 2.9. Cell Culture Maintenance of MC3T3-E1 and Viability Evaluation

The mouse calvaria osteoblastic precursor cell line MC3T3-E1 was used as a model system for the in vitro investigation of the viability, proliferation, and osteogenic differentiation in the presence of the Fmoc-Asp-OFm samples. The cells were cultured in alpha-MEM medium supplemented with 10% fetal bovine serum (FBS), 2 mM l-glutamine, 100 μg/mL penicillin/streptomycin, and 2.5 μg/mL amphotericin (fungizone), in a humidified incubator at 37 °C and 5% CO_2_. After reaching 80–90% confluence, the cells were harvested using trypsin/EDTA; 2 × 10^4^ cells were used for viability/proliferation and differentiation assays, respectively, and were seeded onto the sterilized 48-well Corning^®^ plates coated with Fmoc-Asp-OFm thin layer. The medium was changed every three days. For the differentiation experiments, the primary culture medium was supplemented with 50 μg/mL l-ascorbic acid, 10 mM β-glycerophosphate, and 10 nM dexamethasone. All experiments were carried out using cell passages from 9 to 14.

The viability was determined by means of the resazurin-based PrestoBlue™ assay that stains living cells and is reduced to a product that can be measured photometrically. At each experimental time point (days 3, 7, and 10) in the cell culture, 20 μL of PrestoBlue™ reagent diluted in alpha-MEM at a ratio of 1:10 were added to each well and incubated at 37 °C for 60 min. The volume of 100 μL of the supernatants was transferred to a 96-well plate, and the absorbance was measured at 570 and 600 nm in a spectrophotometer (Synergy HTX Multi-Mode Microplate Reader, BioTek, Bad Friedrichshall, Germany). All samples were rinsed with PBS, and a fresh culture medium was added. The metabolic activity of living cells was correlated with cell numbers by means of a calibration curve. All samples were analyzed in quadruplicates.

### 2.10. Cell Adhesion and Morphology

The adhesion and morphology of the MC3T3-E1 cells were observed by means of SEM after 5 days in culture. Seeded scaffolds with 5 × 10^4^ cells per sample were placed in a CO_2_ incubator at 37 °C for 5 days and were then removed and rinsed with PBS, fixed with 4% v/v para-formaldehyde for 30 min, and were dehydrated in increasing ethanol concentrations (30–100% v/v). The scaffolds were finally dried using hexamethyl disilazane, sputter-coated with a 20 nm thick layer of gold (Baltec SCD 050), and observed under SEM (JEOL JSM-6390 LV) at an accelerating voltage of 15 kV.

### 2.11. Osteogenic Response of MC3T3-E1 Pre-Osteoblastic Cells on Fmoc-Asp-OFm Hydrogels

#### 2.11.1. Alkaline Phosphatase Activity (ALP) of Pre-Osteoblasts on Hydrogels

The alkaline phosphatase activity expressed from the pre-osteoblastic cells cultured in direct contact with Fmoc-Asp-OFm hydrogels was determined by means of an enzymatic assay. Cells were cultured for 7, 14, and 21 days in an osteogenic medium, harvested with trypsin-EDTA and collected by centrifugation. Pellets were treated with 100 μL lysis buffer, exposed to two freeze–thaw cycles, and 100 μL of a 2 mg/mL p-nitrophenyl phosphate (pNPP, Sigma, St. Louis, MO, USA) substrate in 50 mM Tris-HCl at pH 10 with 2 mM MgCl_2_ was added to each sample and incubated at 37 °C for 1 h. The reaction was stopped with 50 μL 1 N NaOH, and the optical density (OD) was measured in a Synergy HTX plate reader (BioTek, Winooski, VT, USA) at 405 nm and was correlated to para-nitrophenol concentrations by means of a calibration curve. The enzymatic activity was calculated using the equation (units = nmol p-nitrophenol/min) and normalized to the cell metabolic activity expressed in OD 570/600. All samples were analyzed in quadruplicates of two independent experiments (n = 8).

#### 2.11.2. Calcium Concentration Determination

Calcium deposits are a late marker of osteogenesis, signaling the formation of an extracellular matrix. The O-cresol phthalein complexone (CPC) method allowed us to determine the total calcium concentration in the culture medium. Specifically, CPC reacts with calcium to form a dark-red colored complex whose absorbance was measured at 570 nm, which is proportional to the amount of calcium in the samples. Supernatants were collected every 3 days up to day 21. A total of 10 μL culture medium from each sample was mixed with 100 μL of calcium buffer and 100 μL of calcium dye CPC, 3.36 mmol/L hydroxy-8-quinoline, and 25 mmol/L HCl. The OD of the solutions was measured at 570 nm. All samples were analyzed in quadruplicates of two independent experiments (n = 8).

#### 2.11.3. Determination of the Produced Extracellular Collagen by the MC3T3-E1

The amounts of total collagen produced by the pre-osteoblastic cells in culture were determined by means of the Sirius Red assay (Direct red 80, Sigma-Aldrich, St. Louis, MO, USA). Briefly, 25 μL of culture medium were diluted in H_2_O at a volume of 100 μL, mixed with 1 mL 0.1% Sirius Red Dye, and incubated for 30 min at room temperature. After centrifugation at 15,000 g for 15 min, the pellets were washed with 0.1 N HCl to remove the non-bound stain. The samples were centrifuged and dissolved in 500 μL 0.5 N NaOH. The absorbance was measured in a Synergy HTX plate reader at 530 nm. The absorbance values were correlated to the concentration of collagen type I by means of a calibration curve. All samples were analyzed in quadruplicates of two independent experiments (n = 8).

### 2.12. Statistical Analysis

Statistical analysis was performed using one-way ANOVA and the Friedman non-parametric test in GraphPad Prism version 8 software to evaluate the significance of the differences between Fmoc-Asp-OFm hydrogels samples compare to the H_2_O control. A *p*-value <0.05 was considered significant.

## 3. Results

### 3.1. Formation and Characterization of Fmoc-Asp-OFm Hydrogels

**Figure 1:**
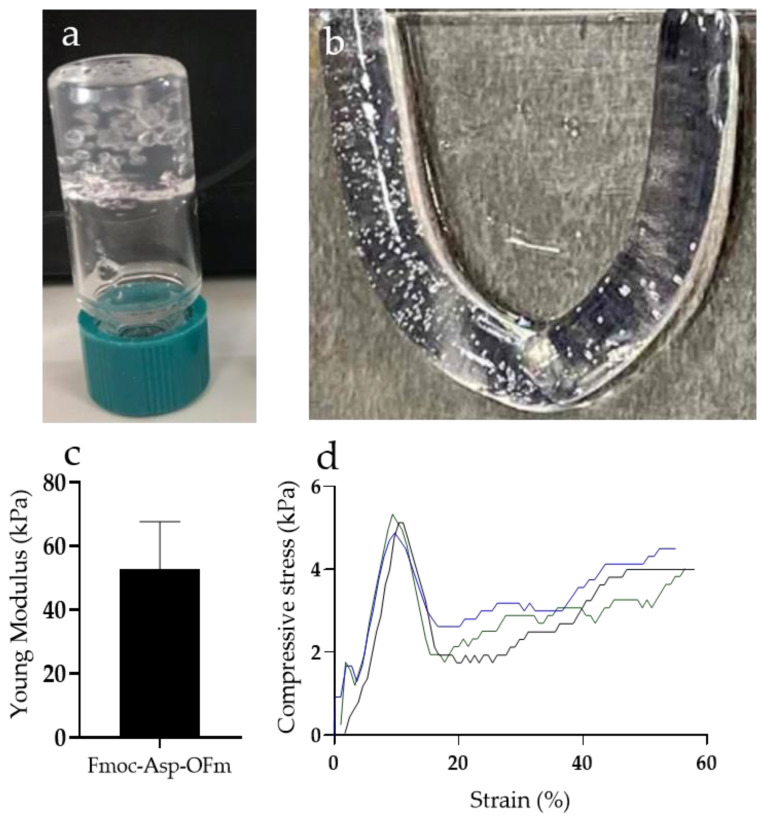
Macroscopic images of self-assembled Fmoc-Asp-OFm (3 mg/mL) hydrogels (**a**) and after syringe extrusion (**b**). Determination of Young modulus for Fmoc-Asp-OFm hydrogels (**c**) and the compressive stress–strain diagram of three measurements (curves shown in different colors) (**d**).

Self-assembly and gelation of the Fmoc-Asp-OFm material were carried out using the solvent-switch method [32]. After the complete dissolution of the peptide in DMSO (termed “good solvent”), H_2_O was used as the second solvent (termed “bad solvent”) to trigger self-organization. Fmoc-Asp-OFm presented great solubility in DMSO while being insoluble in H_2_O. Samples left overnight to mature led to the formation of a fairly stable hydrogel (Figure 1a). The formation of this self-assembled stable hydrogel was extremely straightforward, and the gel could be produced into different geometric morphologies depending on the vessel in which the assembly process was carried out. Figure 1b demonstrates the extrudability of the gel through a syringe after the assembly was carried out in a syringe. This property makes this gel an attractive biomaterial for biomedical applications such as tissue engineering, as it can be injected into a defective tissue.

The Young modulus (or elastic modulus) is a measure of the stiffness of a material. To study the biomechanical characteristics of the produced hydrogel, the Young modulus was measured using compression testing equipment. Self-assembled Fmoc-Asp-OFm hydrogels revealed an average Young modulus of 50 kPa (Figure 1c), which is higher than the elasticity modulus of stiff cartilage [E ≈ 20 to 30 kPa at the scale of adhesions [33,34] and precalcified bone (25–40 kPa) [35]. It can be clearly recognized from the compressive stress strain curves that the Fmoc-Asp-OFm hydrogels present an upper yield stress point at 5% strain which corresponds to approximately 1.8 kPa and an ultimate stress point at 12% strain which corresponds to 5 kPa (Figure 1d).

**Figure 2:**
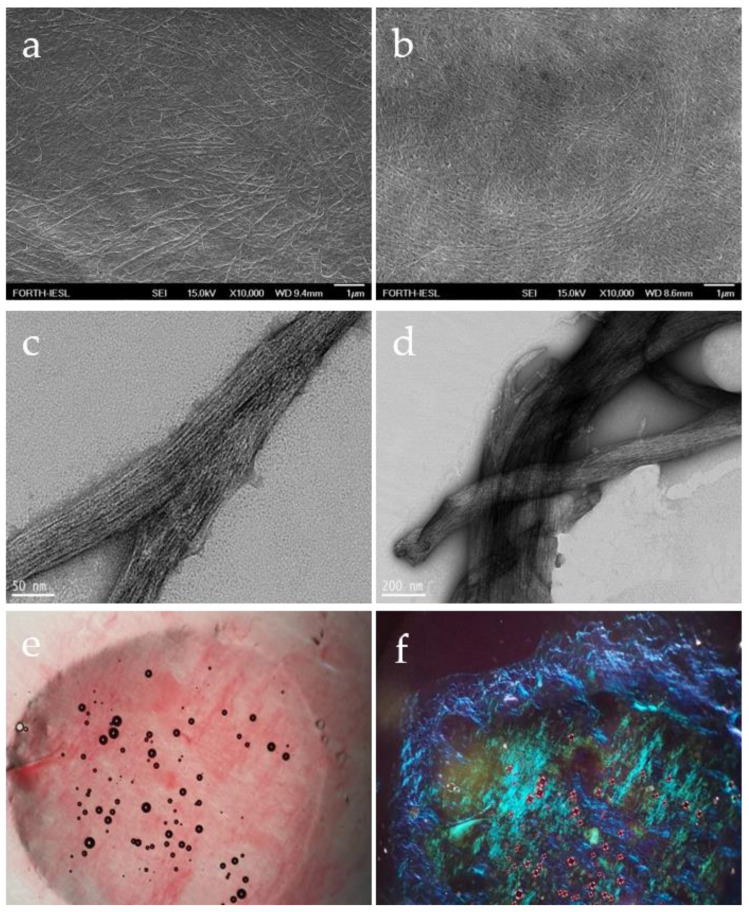
Self-assembly of Fmoc-Asp-OFm (3 mg/mL) depicted by means of FESEM after 1 and 7 days of formation (**a** and **b**, respectively). Different magnification TEM images of Fmoc-Asp-OFm nanofibers (**c**,**d**). Stereoscopic evaluation of amyloid fiber formation via Congo red stain with bright field (**e**) and cross polarizers (**f**).

Field emission scanning electron microscopy (FESEM) revealed the presence of entangled fibrils following gelation, a characteristic of supramolecular hydrogels. The preservation of the self-organization of the fibrils is observed from day 1 (Figure 2a) until day 7 (Figure 2b). A denser fibrillar network is observed after 7 days of formation, indicating their continuous self-organization within this time period. Their parallel arrangement is also evident.

Transmission electron microscopy (TEM) has been used to analyze the molecular organization of the hydrogel and revealed that the Fmoc-Asp-OFm hydrogel consisted of a fibrous network with fibril diameters ranging from 50 to 200 nm (Figure 2c,d).

We then applied the Congo red staining method combined with polarization microscopy to assess the amyloid nature of the Fmoc-Asp-OFm fibrils. Amyloid fibrils, in general, bind Congo red and exhibit a gold/green birefringence under polarized light [10]. Self-assembled Fmoc-Asp-OFm hydrogels bound Congo red (Figure 2e) and exhibited the characteristic green/gold/blue birefringence under polarized light microscopy (Figure 2f).

### 3.2. Ca^2+^ Mineralization on Fmoc-Asp-OFm Hydrogels Formed in CaCl_2_ Solution

The capacity of bone-substituting materials to induce mineralization is often referred to as bioactivity, implying that these materials have the ability to enhance the nucleation of calcium phosphate crystals. Most polymeric materials do not have this ability, but the addition of ceramic materials makes the resulting composites bioactive by providing nucleation sites for the precipitation of hydroxyapatite (HA). The combination of a hydrogel with an inorganic material is inspired by the composite nature of native bone. The addition of a dispersed inorganic mineral may provide nucleation sites for the formation of HA and promote cell adhesion enabling integration with the surrounding tissue [36]. Therefore, inspired by the biomineralization prosses in vivo, we utilized a biomimetic method for the mineralization of the Fmoc-Asp-OFm hydrogels by the 24 h formation of the peptide hydrogel into CaCl_2_ solution.

**Figure 3:**
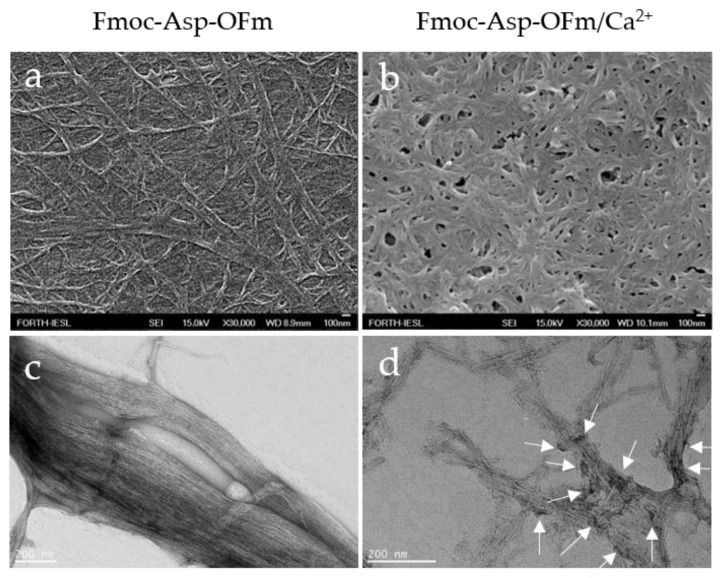
Evaluation of Ca^2+^ binding on Fmoc-Asp-OFm. FESEM images of the hydrogels formed in H_2_O (**a**) compared to the one formed in CaCl_2_ solution (**b**). TEM images of gels formed without calcium (**c**) and in the presence of calcium (**d**). Arrows in (**d**) point to dark nodules of higher electron density that most probably correspond to calcium deposits.

The unmineralized hydrogels presented interconnected pore structures (Figure 3a). After Ca^2+^ mineralization, the hydrogel maintained its porous structure (Figure 3b), but the average pore size significantly decreased due to the direct deposition of the mineral onto the pore walls.

In TEM imaging, the fibrils of the hydrogel that were formed in the absence of calcium were arranged in a parallel manner (Figure 3c), while in the presence of calcium, they displayed a rather interconnected morphology with dark nodules of high electron density, probably corresponding to calcium deposits on the Fmoc-Asp-OFm fibrils (Figure 3d).

**Figure 4:**
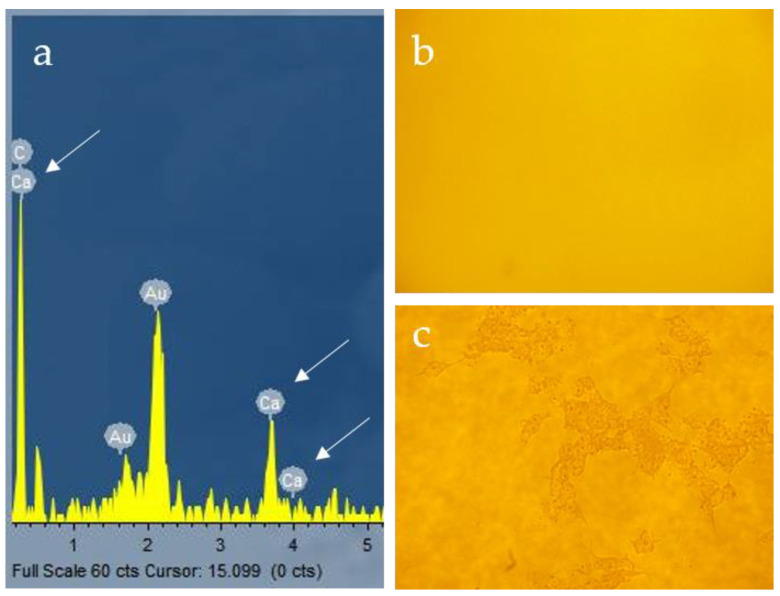
Evaluation of calcium mineralization by means of EDS (**a**). Arrows in spectrum (**a**) indicate the Ca^2+^ peaks. Alizarin red staining of hydrogels formed in the absence (**b**) and in the presence of CaCl_2_ solutions (**c**) confirm the stained calcium deposits in (**c**).

Ca^2+^ mineralization on Fmoc-Asp-OFm/Ca^2+^ was also confirmed with Energy-Dispersive X-ray spectroscopy (EDS) in Figure 4a by the peaks of calcium (Ca) elements in the spectra.

Positive Alizarin red stain and mineral nodule formation were observed in Fmoc-Asp-OFm/Ca^2+^ hydrogels when formed in 7 mM CaCl_2_ (Figure 4c). The calcified nodules appeared bright red in color, indicating that the CaCl_2_ solution could stimulate the formation of calcified nodules significantly when compared to the control in H_2_O (Figure 4b).

### 3.3. Calcium Phosphate Deposition

To induce the formation of calcium phosphate inside the hydrogel matrix, we subsequently immersed Fmoc-Asp-OFm/Ca^2+^ hydrogels in a Na_2_HPO_4_ solution.

**Figure 5:**
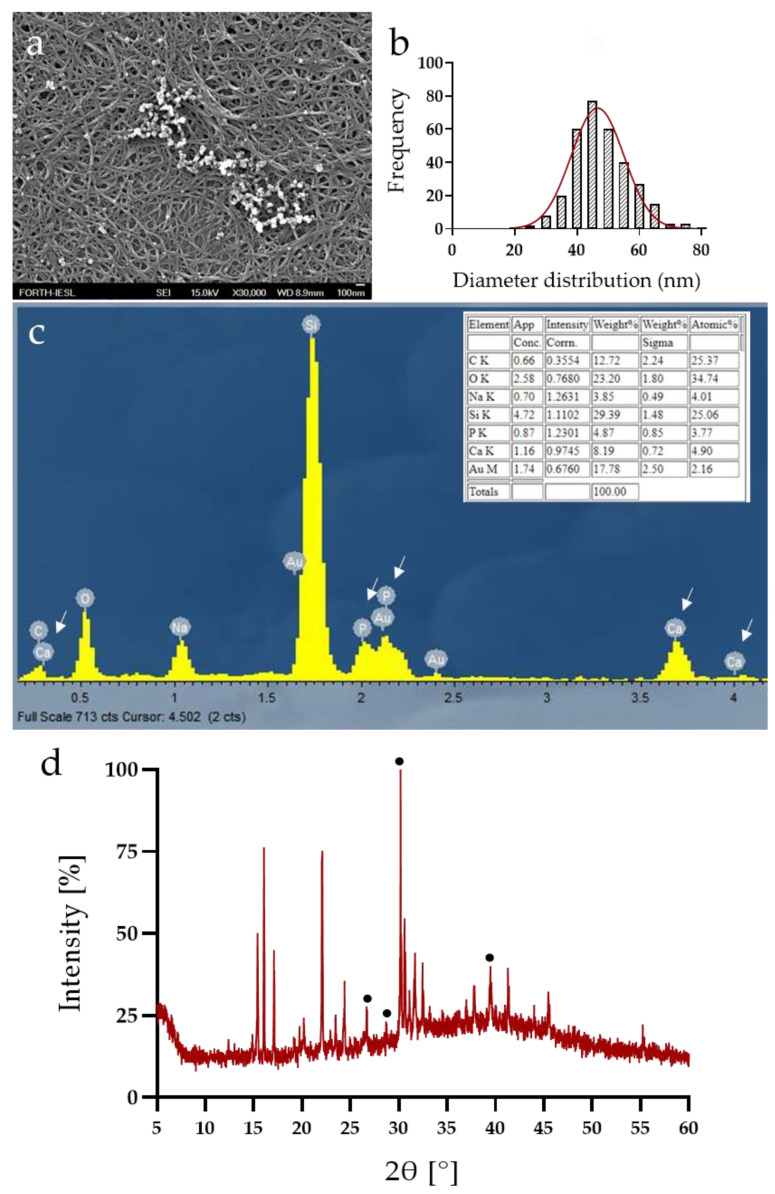
Evaluation of the calcium phosphate deposition on Fmoc-Asp-OFm formed in CaCl_2_ solution after HPO_4_^2−^ deposition by means of FESEM (**a**). Nanoparticle diameter distribution is determined using analysis of FESEM images (**b**). EDS analysis (**c**), the arrows in the spectrum indicate the calcium and phosphorus peaks. XRD diffractogram of mineralized Fmoc-Asp-OFm/Ca^2+^/HPO_4_^2−^ samples with dots displaying characteristic hydroxyapatite peaks (**d**).

To evaluate the deposition process of the mineral phase, we examined the interior morphology of mineralized hydrogels via FESEM. Newly formed Fmoc-Asp-OFm/Ca^2+/^HPO_4_^2−^ hydrogels also presented porous structures (Figure 5a), and similar topography with Fmoc-Asp-OFm and the formation of nanocrystals was also evident. Representative FESEM images depict that all mineralized nanoparticles have a near-spherical shape and tend to form agglomerates. The particle diameter distribution shows that particles have an average diameter of approximately between 30 and 60 nm with a peak at 45 nm (Figure 5b).

EDS data revealed the presence of Ca and P elements as major components in the samples (Figure 5c), confirming calcium phosphate deposition. The Ca/P ratio calculated directly from the EDS results at the value of 1.68, relatively close to the theoretical value (1.67), was characteristic of the stoichiometric hydroxyapatite [37,38].

Inorganic hydroxyapatite synthesis was confirmed by X-ray diffraction analysis, and the obtained diffractogram can be observed in Figure 5d. The spectrum of X-ray diffraction of the prepared xerogel is in good agreement with the reference model of pure hydroxyapatite (JCPDS no. 09-0432). Specifically, the peaks at approximately 26°, 32°, and 40° (2θ) present in the diffractograms correspond to the hydroxyapatite phase. Characteristic peaks of impurities, such as calcium hydroxide and calcium phosphate, have not been observed [39,40].

Based on the above, the diffusion of HPO_4_^2−^ into the Fmoc-Asp-OFm/Ca^2+/^HPO_4_^2−^ hydrogel that contained Ca^2+^ induced calcification of the hydrogel matrix resulting in the formation of calcium phosphate nanocrystals, resemble nanohydroxyapatite formation in vivo.

### 3.4. In Vitro Evaluation of Pre-Osteoblastic Cell Adhesion, Viability and Osteogenic Response of Pre-Osteoblastic Cells on Fmoc-Asp-OFm Hydrogels

**Figure 6:**
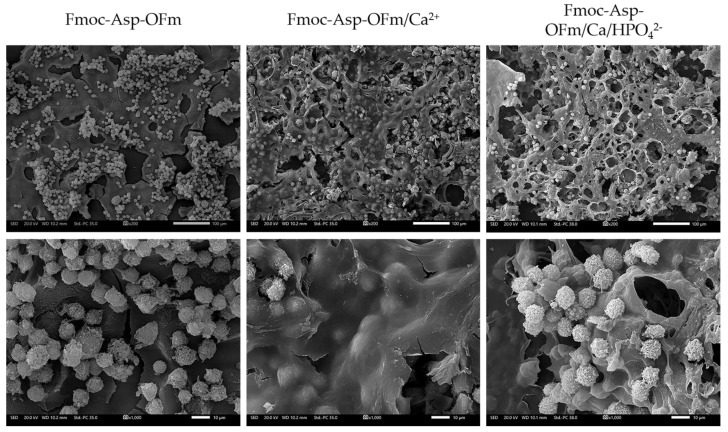
Representative SEM images visualizing the cell adhesion and morphology on Fmoc-Asp-OFm, Fmoc-Asp-OFm/Ca^2+^ and Fmoc-Asp-OFm/Ca/HPO_4_^2−^ hydrogels after 5 days in culture (upper panel at 200× magnification, lower panel at 1000× magnification).

Representative SEM images show pre-osteoblastic cells on Fmoc-Asp-OFm/Ca^2+^, Fmoc-Asp-OFm/Ca/HPO_4_^2−^ and Fmoc-Asp-OFm after 5 days in culture at two magnifications (Figure 6). The effect of surface texture on cell guidance is well known, but how it affects bioactivity is still a subject of investigation [41]. Although the cells do not represent their characteristic elongated morphology on all three types of hydrogels, they display protrusions for cell–cell interactions that are expected to promote tissue formation. Cell infiltration within the pores of the Fmoc-Asp-OFm/Ca^2+^ and Fmoc-Asp-OFm/Ca/HPO_4_^2−^ hydrogels was observed in the lower magnification images of Figure 6 (upper panel).

**Figure 7:**
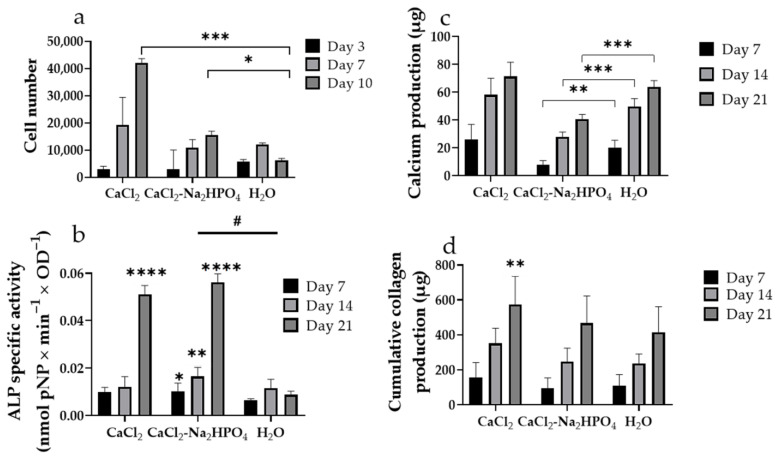
Cell viability and proliferation assessment indicating the cell number of pre-osteoblastic cells seeded on hydrogels formed in CaCl_2_, CaCl_2_-Na_2_HPO_4_ solution, and H_2_O after 3, 7, and 10 days (**a**). Normalized by cell viability (OD values) ALP activity of the pre-osteoblastic cells cultured on the peptide hydrogels for 7, 14, and 21 days (**b**). Calcium production levels from the pre-osteoblastic cells after 7, 14, and 21 days in culture (**c**). Determination of cumulative collagen production by the pre-osteoblastic cells until days 7, 14, and 21 (**d**). Statistical analysis was performed for each composite compared to the control sample formed in H_2_O, using one-way ANOVA (bars with asterisks *) and the Friedman non-parametric test (bar with hash #) (* *p* < 0.05, ** *p* < 0.01, *** *p* < 0.001, **** *p* < 0.0001).

The cell viability and proliferation of the pre-osteoblastic cells in the different hydrogels was quantitatively assessed after 3, 7, and 10 days in the culture. Both hydrogel types formed in CaCl_2_ and CaCl_2_-Na_2_HPO_4_ present a gradual rate of increase in cell numbers over an incubation period of 10 days. Furthermore, both samples exhibit no significant differences compared to the control until day 7, but they seem to induce cell proliferation for longer time periods, as presented in Figure 7a. At day 10, we observed a statistically significant increase in cell population, 7-fold for the CaCl_2_ samples and 2.5-fold for the CaCl_2_-Na_2_HPO_4_ samples compared to the control, indicating an upregulated proliferation potential of MC3T3-E1 cells on the Fmoc-Asp-OFm hydrogels formed in dimethyl sulfoxide DMSO/CaCl_2_.

The ALP activity has been used as an early marker, and the calcium deposition as a late marker for osteogenesis to assess the effect of the hydrogels on the differentiation potential of the pre-osteoblasts to mature osteoblasts. Additionally, the secretion of the total collagen as the main structural component of the extracellular matrix (ECM) was quantified in order to investigate the potential of the pre-osteoblasts cultured on the hydrogels to promote ECM formation. Figure 7b depicts an upregulation of the ALP activity for both CaCl_2_ and CaCl_2_-Na_2_HPO_4_ hydrogels in a comparable manner, with slightly enhanced ALP activity levels from day 7 to day 14, followed by a five-fold increase on day 21. Compared to the control hydrogel in H_2_O with the peak for the ALP activity on day 14, the hydrogel formation in the CaCl_2_ solution resulted in an extension of the ALP expression period and, consequently, an early mineralization phase. The CaCl_2_-Na_2_HPO_4_ hydrogels significantly induced the ALP activity at all the experimental time points, as evidenced by both statistical analyses, the one-way ANOVA, and the Friedman non-parametric test. Regarding the calcium concentration, an increase from day 7 up to day 14 has been observed, which implies that the pre-osteoblasts constantly differentiate into osteoblasts for three weeks in culture (Figure 7c). The amount of calcium measured in the supernatants indicates the highest values for the hydrogels formed in CaCl_2_. Significantly lower levels of calcium in the hydrogels formed in CaCl_2_-Na_2_HPO_4_ can be attributed to the binding of calcium ions with the phosphate groups. Both the ALP activity and the calcium production results present a similar trend regarding the differentiation potential of the Fmoc-Asp-OFm hydrogels formed in CaCl_2_ and CaCl_2_-Na_2_HPO_4_ solutions. The deposition of the type I collagen-rich ECM is required for the expression of specific products, such as the alkaline phosphatase, during the normal developmental sequence of osteoblasts [42]. Regarding the collagen levels secreted by pre-osteoblasts (Figure 7d), both peptide hydrogels demonstrate higher, still comparable values, with the control until day 14. On day 21, the CaCl_2_ hydrogels demonstrated significantly higher collagen levels. These results suggest that both hydrogels support the ECM formation, which is a crucial step required for osteogenic differentiation. As observed, hydrogel formation in the presence of CaCl_2_ seems to positively affect the overall osteogenic capacity of the Fmoc-Asp-OFm hydrogels.

## 4. Discussion

Hard tissues such as bone are mineralized tissues with a high degree of hardness [43]. Bones present a good self-healing ability after fracture or defect [44]. Several biomaterials, such as metal implants, allografts, autografts, ceramics, and composites, have been applied for bone tissue regeneration [45,46,47]. Although biomaterials such as ceramics, bio-glasses, and bone cement are crucial for bone tissue repair, they are insufficient in promoting self-tissue regeneration in the bone. Hence, the development of a new generation of biomaterials that serve as templates for hard tissue growth is a growing field [48,49]. In this work, we report on the Fmoc-Asp-OFm self-assembling fibrous structures as biomimetic hydrogel matrices with calcium ion binding sites that act as nucleation points for phosphate anions for bone tissue engineering applications.

The self-assembly of this minimal building block led to the formation of amyloid-type fibrils, as assessed by TEM, SEM, and Congo Red staining. The Fmoc-moiety plays a critical role in the self-assembly process through aromatic stacking interactions, and its capacity to induce gelation is also well-established. When the hydrogels were allowed to assemble in the presence of calcium chloride, calcium was detected with EDS analysis after the immersion of the gel matrix in water and exhaustive washing. This indicates that aspartic acid can capture calcium ions onto the fibril framework. Following the addition of dibasic calcium phosphate ions onto the calcium matrix, both calcium and phosphorus could be detected by EDS analysis, suggesting that the calcium ion sites can attract phosphate ions and serve as nucleation templates, resulting in calcium phosphate deposits. X-ray diffraction analysis of freeze-dried hydrogels revealed that their formation in the presence of calcium and phosphate ions results in a composite material in which its inorganic compound represents a pure hydroxyapatite phase.

Recently, there has been significant interest in developing injectable hydrogel scaffolds for minimally invasive implantation procedures toward the efficient healing and regeneration of defective and damaged bone tissue. Hydrogels offer several benefits due to their 3D hydrophilic nature and their porous network structure with the ability to absorb considerable amounts of water or body fluids simulating the ECM of the host bone and thereby facilitating cell proliferation [50,51]. In addition to mimicking the in vivo environments of ECM, hydrogels can be easily injected into any irregular bone fracture and cause minimal mechanical irritation to the surrounding tissue [52,53]. In our study, amino acid-based material is attached to protecting groups and self-assembles into biocompatible and stable nanostructures. The Young’s modulus of self-assembled Fmoc-Asp-OFm hydrogel scaffolds exceeded the value of 50 kPa, which is ten-fold higher compared to other natural hydrogels reported in the literature [54,55]. Peptide hydrogels were reported to reach Young’s Modulus as high as 50 kPa [18]. It is noted that while the resulting mechanical properties of the investigated hydrogels are inferior to the native calcified bone interface [56], a successful combination of cell-mediated matrix deposition and scaffold mechanical properties dictates the regeneration of functional tissues that can be formed onto the composite hydrogels.

The investigation of suitable biomaterials for bone TE includes their potential to promote cell adhesion, viability, proliferation, and osteogenic differentiation. In this context, the osteogenic potential of the novel self-assembled, mineralized hydrogels was evaluated by direct contact with pre-osteoblastic cells. It is likely that the complete success of pre-osteoblastic cell differentiation in vitro for bone regeneration will require osteoblast binding and differentiation sites, reflecting the transport processes that promote the mineralization of the dense bone matrix [42]. Our results indicate that the elevated extracellular Ca^2+^ leads to cell adhesion, viability, and proliferation increase, which may be attributed to the surface chemistry of an increased surface area as an important mechanism to enhance the interaction between osteoblasts and the crystalline calcium-containing matrices [57]. The induction of calcium phosphate deposition by Na_2_HPO_4_ treatment appears to be associated with a moderate increase in cell proliferation compared to control hydrogels (formed in H_2_O). It should be noted that the mechanisms of interaction between hydrogel networks and the supplemented inorganic particles still need to be elucidated in depth. Data on prolonged in vivo applications of such composite hydrogels are very limited, and thus, further comprehensive studies on their long-term performance, including biocompatibility, biodegradability, and osteogenic activity in vivo, are needed to confirm the promising properties of this class of materials for bone tissue engineering [58].

To further investigate the potential of the developed composite hydrogels to serve as a scaffold that can induce osteoblast differentiation, we measured the ALP specific activity, calcium, and collagen production of the MC3T3-E1 osteoblast precursors. Intramembranous ossification is the process of bone formation from fibrous membranes, through which bone tissue develops by the concentration of pre-osteoblastic cells that directly undergo osteogenic differentiation [59,60]. During intramembranous ossification, osteoblasts deposit bone matrixes through the production of collagen type I fibrils and the regulation of deposited minerals onto the collagenous matrix [60]. For the regulation of the mineralization of the collagenous matrix, osteoblasts express proteins, including the ALP, which provides the phosphate groups required for the mineralization [61]. In the present study, pre-osteoblasts exposed to the mineralized hydrogels formed in the presence of CaCl_2_ and CaCl_2_-Na_2_HPO_4_ indicate an increase in the ALP activity, elevated calcium levels, and support the collagen production for the ECM formation, which is a crucial step required for osteogenic differentiation. Other groups have described an osteoconductive [62] and osteoinductive [63,64] effect of calcium phosphate minerals on osteoblast precursors cells. Consequently, the presence of a mineralized matrix, as a result of the Fmoc-Asp-OFm hydrogels mineralization, could stimulate the pre-osteoblastic cells into an osteoblastic phenotype. Furthermore, the fibrillar morphology of the composite self-assembled hydrogel and the 3D environment allowed the diffusion of osteogenic stimuli to the adhered cells, thus promoting their differentiation into osteoblasts.

The cell viability, proliferation, and osteogenic differentiation of pre-osteoblastic cells cultured on the functional hydrogels under various conditions demonstrate that hydrogel formation in CaCl_2_ and CaCl_2_-Na_2_HPO_4_ solutions lead to calcium ion binding onto the hydrogels and enrichment with phosphate groups, respectively, rendering these mechanically stable hydrogels osteoinductive scaffolds for bone tissue engineering.

## 5. Conclusions

Τhis study represents a successful attempt to assess the suitability of a single amino acid-based hydrogel for bone tissue regeneration. For this, we used as the monomolecular building block a doubly Fmoc-protected aspartic amino acid that self-assembles into amyloid-type structures and forms stable and stiff hydrogels. The aspartic acid moieties enable the hydrogel network to act as a framework for templating calcium ions that subsequently serve as nucleation sites for phosphate ions. This resulted in a composite bioactive, self-assembled hydrogel matrix for the promotion of hydroxyapatite precipitation. The hydrated composite matrix creates an osteoinductive material that promotes the adhesion, viability, and proliferation of pre-osteoblastic cells. In addition, the differentiation of pre-osteoblastic cells into mature osteoblasts revealed that the bioinspired mineralized hydrogels promoted their osteogenic capacity. The enhanced mechanical strength, the extrudability of the hydrogels, and the 3D deposition of bone apatite in the cell-laden composites make these protected amino acid-based hydrogels promising candidates for bone tissue engineering. Their minimalistic nature and relatively low cost, combined with commercial availability, could pave the way for their wider applicability in hard tissue engineering.

## Figures and Tables

**Table 1:** Sample abbreviations and compositions.

Name	Composition and Formation Conditions
Fmoc-Asp-OFm	e.g., Control Fmoc hydrogel 3 mg/mL Fmoc-Asp-OFm dissolved in DMSO and formed in bi-distilled water (2:8)
Fmoc-Asp-OFm/Ca^2+^	3 mg/mL Fmoc-Asp-OFm dissolved in DMSO and formed in 7 mM calcium chloride (CaCl_2_) solution
Fmoc-Asp-OFm/Ca/HPO_4_^2−^	3 mg/mL Fmoc-Asp-OFm dissolved in DMSO and formed in 7 mM calcium chloride and 0.6 M dibasic sodium phosphate (Na_2_HPO_4_) solution

## Data Availability

Not applicable.

## Supplementary Materials

The following supporting information can be downloaded at: https://www.mdpi.com/article/10.3390/ma15248928/s1, Figure S1: Casting process and macroscopic images of Fmoc-Asp-OFm hydrogels.
