# Supplementary material for: A Doubly Fmoc-Protected Aspartic Acid Self-Assembles into Hydrogels Suitable for Bone Tissue Engineering
Source: Materials (Basel). 2022 Dec 14;15(24):8928. doi: 10.3390/ma15248928 (PMC9784766; doi:10.3390/ma15248928)
Supplement: Supplementary file 1 [file materials-15-08928-s001.zip › materials-2048501-supplementary.pdf]

materials

Electronic Supporting Information for:

A Doubly Fmoc-Protected Aspartic Acid Self-Assembles into Hydrogels Suitable for Bone Tissue Engineering

Katerina Petropoulou [1,†], Varvara Platania [2,†], Maria Chatzinikolaidou [2,3,*] and Anna Mitraki [2,3,*]

1 Department of Biology, University of Crete, 70013 Heraklion, Greece; katerinapetr1302@gmail.com
2 Department of Materials Science and Technology, University of Crete, 70013 Heraklion, Greece; vplatania@materials.uoc.gr
3 Institute of Electronic Structure and Laser (IESL), Foundation for Research and Technology Hellas (FO.R.T.H), 70013 Heraklion, Greece
* Correspondence: mchatzin@materials.uoc.gr (M.C.); mitraki@materials.uoc.gr (A.M.)
† These authors contributed equally to this work.

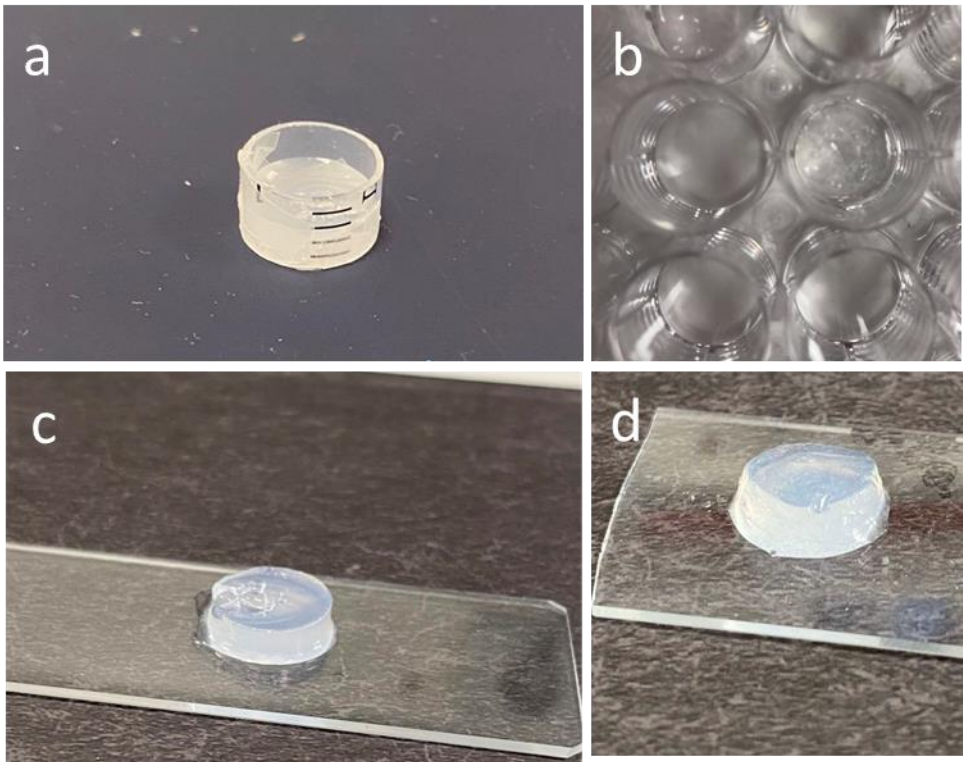

Figure S1. Casting process and macroscopic images of Fmoc-Asp-OFm hydrogels. Fmoc-Asp-OFm powder (3 mg/ml) was dissolved in DMSO and formed in a 7 mM $CaCl_2$ solution. Creation of a Fmoc-Asp-OFm/Ca^{2+} hydrogel in a cut syringe barrel (a), washing of a Fmoc-Asp-OFm/Ca^{2+} hydrogel in 24 well-plates (b), hydrogel before washes (c), hydrogel after washes (d).
